# Study protocol: health survey of Sao Paulo: ISA-Physical Activity and Environment

**DOI:** 10.1186/s12889-021-10262-5

**Published:** 2021-02-04

**Authors:** Alex Antonio Florindo, Inaian Pignatti Teixeira, Ligia Vizeu Barrozo, Flávia Mori Sarti, Regina Mara Fisberg, Douglas Roque Andrade, Leandro Martin Totaro Garcia

**Affiliations:** 1 School of Arts, Sciences and Humanities, University of Sao Paulo, Rua Arlindo Bettio, 1000, Sao Paulo, SP 03828-000 Brazil; 2 Graduate Program in Nutrition in Public Health, School of Public Health, University of Sao Paulo, Sao Paulo, Brazil; 3 Physical Activity Epidemiology Group, University of Sao Paulo, Sao Paulo, Brazil; 4 Department of Geography, School of Philosophy, Literature and Human Sciences, University of Sao Paulo, Sao Paulo, Brazil; 5 Department of Nutrition, School of Public Health, University of Sao Paulo, Sao Paulo, Brazil; 6 Centre for Public Health, Queen’s University Belfast, Belfast, UK

**Keywords:** Built environment, Leisure-time physical activity, Active travel, Cohort, Adults

## Abstract

**Background:**

Many studies have investigated the association between the built environment and physical activity behavior in urban settings. However, most of the studies conducted in low- and middle-income countries were cross-sectional, which are limited to identify behavioral determinants. We propose a prospective cohort study to verify the relationship between built environment features and leisure-time and transport-related physical activity in adults from Sao Paulo city, Brazil.

**Methods:**

Prospective multilevel cohort, denominated “ISA-Physical Activity and Environment”. It will build on the Health Survey of Sao Paulo in 2015 (“*Inquérito de Saúde de São Paulo (ISA)*” in Portuguese). The Health Survey of Sao Paulo, originally designed as a cross-sectional survey, had a multi-stage sample, covering 150 census tracts distributed in five health administrative areas. Data collection was performed by face-to-face interviews until December 2015 and the sample comprised 4043 individuals aged 12 years or more. The ISA-Physical Activity and Environment study will reassess people who are aged 18 years or more in 2020, including telephone and household interviews. The primary outcome will be leisure-time and transport-related physical activity, assessed through the International Physical Activity Questionnaire long version. Exposure variables will be built environment features in the areas participants live and work in the follow-up. Data analysis will include multivariate multilevel linear and logistic models. We will also conduct cost-effectiveness analysis and develop agent-based models to help inform decision-makers. The study will be conducted by an interdisciplinary research team specialized in physical activity epidemiology, nutritional epidemiology, georeferencing applied to health, statistics, agent-based modeling, public health policy, and health economics.

**Discussion:**

There are few longitudinal studies on the relationship between the built environment and physical activity behavior in low- and middle-income countries. We believe that the ISA-Physical Activity and Environment study will contribute with important results for the progress of the knowledge in this field and for the implementation of policies that promote leisure-time physical activity and active travel in Sao Paulo and similar cities across the world.

## Background

Leisure-time physical activity and active transportation are behaviors significantly associated with the prevention of chronic non-communicable diseases and the promotion of healthier environments. Cities that encourage physical activity among their inhabitants usually present important results in the reduction of greenhouse gas emissions and motorized traffic, improvement in social capital, and an increase of green areas [1–7]. However, studies addressing physical activity behavior among adults in Latin American countries presented varied results throughout the last decades.

A survey involving more than 370 thousand adults in Brazilian states’ capitals from 2006 to 2012 showed that there was a 2% annual increase in the proportion of people doing at least 150 min of leisure-time physical activity. However, this study showed a larger decrease in active transportation: 13% per year between 2006 and 2008 and 6% between 2009 and 2012 [8]. Another recent study conducted in Sao Paulo city, Brazil, including more than 21 thousand adults, showed a 7.9 p.p. increase in exercise or sports in leisure time from 2006 to 2016 [9]. However, in Colombia, a study involving more than 27 thousand adults between 2005 and 2010 indicated a reduction in leisure-time physical activity, although there was an increase in walking for transportation [9].

The impact of physical activity on health is well-established, as well as the fact that physical activity is affected by complex multidimensional factors that can hinder or facilitate behavioral changes for more active lifestyles worldwide. A study published in the Lancet series on physical activity showed more than 70 characteristics associated with physical activity behavior in populations from low- and middle-income countries [10], a large number of these features are associated with cities’ built and natural environment. Cities’ environmental characteristics refer to physical features such as patterns of land use, street connectivity, and number and diversity of destinations reachable by walking or cycling from households, including parks, squares, clubs, gyms, bicycling paths, schools, and public transportation [11].

However, most of the studies on the role of cities’ built environment on leisure-time and transport-related physical activity have been conducted in high-income countries, which present socioeconomic and cultural aspects that are different from low- and medium-income countries [12]. Besides, most studies published in low- and middle-income countries are based on cross-sectional data, limiting their capacity to answer whether different built environment features can affect physical activity behavior [12]. A systematic review published in 2012 indicated that 89% of the studies identified a significant association between features from the built environment and physical activity level in diverse populations [13].

Epidemiologic studies conducted in Brazil from 2010 onwards showed that people living within buffers of 500 m to 1500 m with a higher density of bicycling pathways, parks, gyms, subway or train stations, improved walkability, and mixed destinations, had a higher probability of engaging in leisure-time and transport-related physical activity [2, 14–18]. These studies were important to identify potential associations between exposure to certain built environment features and physical activity behavior; however, they present limitations and uncertainty regarding trends and causality [19]. For instance, it is uncertain whether individuals were already physically active and therefore chose to live in environments with improved infrastructure, or whether neighborhoods with better built environment features contributed to enhancing physical activity levels.

Few longitudinal studies have been proposed to assess potential causal links between the built environment and physical activity level in high-income countries [20–22], and even less in low- and middle-income countries. The lack of longitudinal data in large multicultural middle-income countries like Brazil is an important knowledge gap that needs to be addressed, particularly considering recent changes in Brazilian society. It is the case of Sao Paulo, the largest city in Brazil, with more than 12 million inhabitants and one of the ten largest cities worldwide, which had an urban masterplan approved in 2014 targeting environmental inequities to improve the quality of life of its residents.

Therefore, additional efforts in the analysis of individual, environmental and social determinants and correlates of physical activity are required in developing countries, especially considering the potential post-COVID-19 developments, which may have long-term impacts on physical activity levels.

### Aims of the study

The “Health Survey of Sao Paulo: Physical Activity and Environment” (*“Inquérito de Saúde de São Paulo-ISA: Atividade Física e Ambiente”* in Brazilian Portuguese) is a prospective cohort study in Sao Paulo city, Brazil, focusing on the evolution and the association between built environment features of the areas where people live and work and leisure-time physical activity and active travel. Other secondary objectives include the analysis of the relationship between the built environment and sedentary behavior, nutritional status, mobility, the incidence of diseases, and mental health.

## Methods

### Study design

A prospective multilevel cohort of individuals living in the city of Sao Paulo, Brazil.

### Settings and study population

#### Setting

The study will be conducted in Sao Paulo city, Brazil, which has a population of 12,325,232 million inhabitants as of 2020, being the most densely populated region in the country (8102.79 inhabitants per km^2^) and one of the ten most populated cities worldwide. It is organized into 96 districts, grouped in 32 sub-prefectures, six health administrative areas, and, as of 2019, included 27,149 census tracts.

#### Baseline

The longitudinal study builds on the Health Survey of Sao Paulo conducted in 2015 as its baseline. The Health Survey of Sao Paulo 2015 was a cross-sectional health survey coordinated by researchers from the University of Sao Paulo, Campinas State University, and Health Institute of Sao Paulo, in partnership with the Sao Paulo Municipal Secretary of Health. The survey aimed at providing data on prevalence and potential correlates of the population’s health and lifestyle, generating evidence for public policies in the city.

The sample design and methodological procedures of the Health Survey of Sao Paulo 2015 were published elsewhere [23]. Briefly, the sample design had two levels: (1) census tracts; and (2) households. In the first stage, 150 census tracts across five health administrative areas (30 census tracts per area) were selected. In the second stage, visits were conducted in 5469 households: 18.3% in the North region, 21.8% in the Middle-West region, 21.8% in the Southeast region, 20.5% in the South region, and 17.6% in the Southeast region. Face-to-face interviews were conducted in households with 4043 individuals aged 12 years or more from August 2014 to December 2015.

#### ISA-Physical Activity and Environment study

**Fig. 1:**
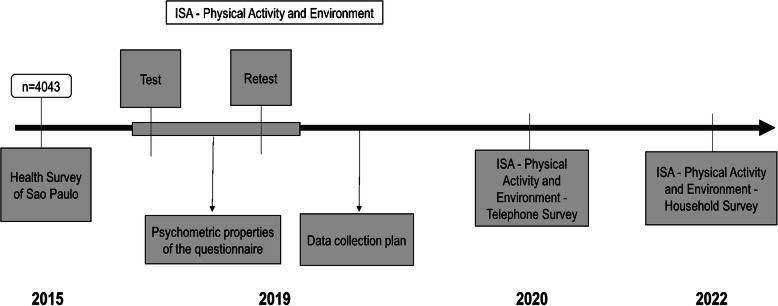
Design of the ISA-Physical Activity and Environment Study

For the longitudinal study, participants of the Health Survey of Sao Paulo 2015 who are aged 18 years or more at the time of the new interview will be invited to participate in the follow-up. Data collection will be done in household visits and telephone interviews (Fig. 1).

Considering the restrictions imposed after the COVID-19 pandemics, the initial follow-up interview during 2020 will be performed through telephone, adopting the standard methodology used in the Surveillance of Risk and Protection Factors for Chronic Diseases by Telephone Survey (VIGITEL), from the Brazilian Ministry of Health [24]. The method is based on computer-assisted telephone interviewing (CATI), which is conducted by an interviewer using a computer to complete the survey directly in an electronic questionnaire during the telephone call. At the end of the interview, the answers are readily available within the survey dataset for analysis. Other data collection stages will be performed in 2022.

#### Sampling power for longitudinal analysis

Cohort and natural experiment studies have been able to reassess at least 70% of the baseline sample in intervals of 2 to 3 years [20, 25, 26]. Considering that the main exposure variables in the study refer to the built environment near interviewees’ households, as parameters to calculate sample size we adopted the results of a cross-sectional study performed using the same sample, which focused on the association between access to public open spaces (e.g., parks, squares, and bicycle paths within distances up until 500 m of participants’ households) with leisure walking [17]. The prevalence in the exposed group (individuals who had access to two or more built environment features within 500 m from their household) was 26.9% and prevalence in the non-exposed group was 18.9%. Adopting a significance level of 5 and 90% power for single-tailed hypothesis testing in a cohort study, at least 469 people per exposure group should be sampled.

#### Research team

The study encompasses an interdisciplinary research team specialized in physical activity epidemiology, nutritional epidemiology, georeferencing applied to health, statistics, agent-based modeling, public health policy and health economics, from the University of Sao Paulo (Brazil), other research institutions from the state of Sao Paulo and other Brazilian states, Australia, United Kingdom, Portugal, and United States.

#### Ethical aspects

The ISA-Physical Activity and Environment study was approved by the Ethics Committee of the School of Arts, Sciences and Humanities at the University of Sao Paulo on April 08, 2019 (protocol number 10396919.0.0000.5390).

#### Individual-level data collection

The questionnaires adopted for telephone and household interviews are based on the Health Survey of Sao Paulo. Physical activity level will be assessed using the International Physical Activity Questionnaire (IPAQ) long version [27], standardized to evaluate activities performed in a typical week. The method has been used in previous studies on the associations between the built environment and physical activity in South American countries, and questions about leisure-time and transport-related physical activity are adequate to assess physical activity in these domains in countries like Brazil and Colombia [27].

The reproducibility of the IPAQ was tested in a sample of 43 individuals (mean age = 45.7 years; standard-deviation (sd) = 20.3; range = 18 to 82). The average interval between interviews was 8.2 days (sd = 1.3; range = 7 to 11). Results indicated that there were no statistically significant differences between the first and the second interviews in each of the domains of physical activity, as well as for leisure walking and moderate-to-vigorous activity, and for transport-related cycling and walking. After dichotomization, results from the first and second interviews of each domain and type of physical activity were significantly associated, with an agreement higher than 70% in all domains and types.

The questionnaires include 11 thematic sections in the 2020 telephone interview and 20 thematic sections in the face-to-face interviews from 2022 onwards. The description of variables that will comprise the primary and secondary outcomes in the study are provided in Table 1.

**Table 1:** Dimensions of the questionnaires adopted in the ISA-Physical Activity and Environment study in Sao Paulo city, Brazil

Dimension	Evaluations	
	Wave 2 - Telephone interviews	Wave 3 - Face-to-face interviews
Physical activity	Yes	Yes^a^
Sedentary behavior	Yes	Yes^a^
Perceived environment	Yes	Yes
Self-efficacy for physical activity	No	Yes
Social support for physical activity	No	Yes
Barriers to physical activity	No	Yes
Mobility and transportation	Yes	Yes
Sleep	Yes	Yes^a^
Nutritional status	Yes	Yes
Food consumption	No	Yes
Perceived health status	Yes	Yes
Emotional health	No	Yes
Self-report of diseases	Yes	Yes
Socioeconomic level	Yes	Yes
Household characteristics	Yes	Yes
Physical activity during the COVID-19 pandemic	Yes	No
Obesity during the COVID-19 pandemic	Yes	No
Self-assessment of neighborhood	Yes	Yes

^a^Self-report and accelerometer

#### Accelerometer-based assessment of physical activity

Those sampled to participate in the study in 2022 will receive accelerometers (Actigraph, model GT3X) to be used in the wrist for 24 h per day, for seven consecutive days, to register physical activity, sedentary, and sleep behaviors [28–32].

#### Built environment data collection

A geoprocessing process will be implemented to obtain built environment data using primarily geographic information systems, obtained through an online library of geospatial data publicly available at the Sao Paulo Municipal Government (http://geosampa.prefeitura.sp.gov.br/PaginasPublicas/_SBC.aspx). Other datasets of interest will also be used during the development of the project, considering information from 2014 onwards.

Data referring to infrastructure, such as green spaces (squares, parks, trees), sports facilities (schools with sports infrastructure, sports centers, community clubs, private clubs, private spaces with sports activities, private gyms, open air gyms), facilities for leisure and transportation (bike paths), education facilities (public schools, private schools, social services units, technical schools, and universities), facilities for leisure and culture (theaters, museums, cinemas, libraries, cultural centers, arts galleries), traffic security (traffic lights, volume of vehicles), public security (street lighting, theft rates, homicide rates), public transport (bus stops, train and subway stations, bus terminal), primary health care units, commerce facilities (supermarkets, restaurants, bakeries, coffee shops, open street markets, fast-food restaurants), and other socioeconomic and physical structures (streets connectivity – including stairways and alleys – topography, commerce density, and residential density).

Built environment data will be considered within 500 m, 1000 m and 1500 m radius and network around the participants’ residence and workplace [33, 34]. The standardization adopted in the baseline studies (Health Survey of Sao Paulo 2015) will also be included in the analysis to improve the quality of the information in the reference period [16–18]. The geoprocessing process will be performed using the ArcGIS Desktop software, version 10.8.1.14362, Copyright (C)1999–2020 Esri Inc.

Beyond geospatial data obtained through geographic information systems, audit analysis will be performed using the Microscale Audit of Pedestrian Streetscapes (MAPS), global version [35]. MAPS is a tool that assesses crossings, street segments, and a route up until 0.72 km from the interviewees’ households, auditing aspects regarding land use, presence of malls and shopping centers, street utilization, aesthetics, social aspects, characteristics of streets and sidewalks, intersection controls, street lights, gutters, crosswalks, signaling for bicycles, dead-end streets, squares, and parks. The tool was originally designed for in loco audits; however, studies from different countries have shown that remote audit is equally reliable [36]. Thus, the present study will apply MAPS on data imagery provided through Google Street View, which presents panoramic views of the streets.

#### Interviews with stakeholders

Complementary to the longitudinal study, organizations from the public, private, and non-governmental sectors have been conducted have been engaged since the beginning of the study. in-depth interviews were conducted to know their perspective on the main challenges to promote and increase physical activity in the city, analyze which problems may be addressed in the study, and maximize the potential for using the evidence that will be obtained for implementing public policies and programs that promote leisure-time and transport-related physical activity, according to the premises proposed by Giles-Corti et al. [37] and Leyden et al. [38]. The organizations were selected considering their representativeness in the areas of physical activity and sports, active transportation, urban mobility, public health, education, and urban development in Sao Paulo city, particularly considering the primary outcomes of the research.

#### Cost-effectiveness and agent-based modeling

The study will be followed by an analysis of health impacts and cost-effectiveness of alternative scenarios considering feasible structural changes in the built environment that may influence leisure-time and transport-related physical activity. Data from the survey will be complemented by existing evidence in the literature to propose an agent-based model to simulate daily activities in different neighborhoods and test potential changes in the built environment aimed at the population leisure and transport-related physical activity behavior and to estimate the consequent health impacts of these environmental and behavioral changes. Cost-effectiveness analysis applied to scenarios in the agent-based models that show larger impacts on physical activity levels will be performed for primary outcomes and certain secondary outcomes, as described in Table 2. The results of these two modeling components can then be used to inform potential public policies in infrastructure, health, and leisure in the city.

**Table 2:** Aspects included in the economic assessment component of the ISA-Physical Activity and Environment in Sao Paulo city, Brazil

Components	Outcomes	
Leisure-time physical activity	Changes in walking duration (minutes)	Walk^t1^ – Walk^t0^ $\frac{t_{wl}^{t1}-{t}_{wl}^{t0}}{t_{wl}^{t0}}\times 100$
	Changes in moderate-to-vigorous physical activity duration (minutes)	Mod-Vig^t1^ – Mod-Vig^t0^ $\frac{t_{mv}^{t1}-{t}_{mv}^{t0}}{t_{mv}^{t0}}\times 100$
Active travel	Changes in walking duration (minutes)	Walk^t1^ - Walk^t0^ $\frac{t_{wt}^{t1}-{t}_{wt}^{t0}}{t_{wt}^{t0}}\times 100$
	Changes in bicycling duration (minutes)	Bicycl^t1^ – Bicycl^t0^ $\frac{t_{bt}^{t1}-{t}_{bt}^{t0}}{t_{bt}^{t0}}\times 100$
Nutritional status	Change in Body Mass Index (BMI, in kg/m^2^)	BMI^t1^ – BMI^t0^ $\frac{BMI^{t1}-{BMI}^{t0}}{BMI^{t0}}\times 100$
Individual behavior	Clothing and other accessories for leisure-time walking (clothes, shoes, and specific accessories for walking)	
	Clothing for leisure-time moderate-to-vigorous physical activity (clothes, shoes, and specific accessories for specific activities)	
	Equipment for leisure-time moderate-to-vigorous physical activity (equipment for gymnastics and weight training)	
	Equipment for active travel (bicycle and safety equipment)	
	Equipment maintenance	
	Physical education professionals’ wages	
	The opportunity cost of time spent in leisure walking	
	The opportunity cost of time spent in leisure-time moderate-to-vigorous physical activity	
	The opportunity cost of time spent in active travel	
Built environment	Building and maintenance of leisure facilities (clubs and community centers, parks and squares)	
	Facilities and green spaces for physical activity in public places (trees and equipment for physical activity)	
	Physical education professionals’ wages in public sector programs	
	Building and maintenance of active travel facilities (bicycle paths and sidewalks)	
	Public transport infrastructure (train and subway stations and bus stops)	
	Destination mix (density of commercial, leisure, and food facilities)	
	Changes in land use (differences in values of real state, based on municipal taxes: household and commerce density, commerce/household ratio, street connectivity)	
	Changes in transportation infrastructure (differences in transportation costs, whether individual or collective, concerning the adoption of bicycle or walking)	

Obs.: t_wl_^to^ = duration of leisure walking at baseline; t_wl_^t1^ = duration of leisure walking at follow-up; t_mv_^to^ = duration of moderate-to-vigorous physical activity at baseline; t_mv_^t1^ = duration of moderate-to-vigorous physical activity at follow-up; t_wt_^to^ = duration of transport-related walking at baseline; t_wt_^t1^ = duration of transport-related walking at follow-up; t_bt_^to^ = duration of transport-related bicycling at baseline; t_bt_^t1^ = duration of transport-related bicycling at follow-up; BMI^t0^ = Body Mass Index at baseline; BMI^t1^ = Body Mass Index at follow-up

### Measures

#### Primary outcomes

The main outcomes of this study will be 1. Leisure-time walking; 2. Leisure-time moderate-to-vigorous physical activity; 3. Total leisure-time physical activity (walking, moderate and vigorous activities); 4. Transport-related walking; 5. Transport-related bicycling.

#### Main secondary outcomes

Secondary outcomes include work-related physical activity; household physical activity; sedentary behavior; body mass index; nutritional status; quality of food consumption; mental diseases; respiratory diseases; hypertension; type 2 diabetes; sleep patterns; urban mobility patterns; perceived environment for physical activity; self-efficacy for physical activity; social support for physical activity; exercise and nutritional status during COVID-19 pandemics; socioeconomic level.

#### Data analysis

The estimation of primary outcomes (physical activity levels) and exposures (built environment features) will be based on differences between the baseline survey (conducted until 2015) and follow-up measures.

Multivariate multilevel linear and logistic models will be estimated to consider census tracts, households, and confounding variables. Directed acyclic diagrams will be adopted for the identification and selection of confounding variables for the regression models.

Moderation and mediation analysis will be performed to investigate whether and how the built environment influences physical activity behavior through changes in psychological attributes and the moderating role of certain socio-economic conditions. In addition, data in 2020 will be used to investigate the effects of restrictions due to COVID-19 pandemics on physical activity levels, and changes in body weight.

The cost-effectiveness analysis will be based on the observed effects of changes in the built environment on health outcomes. The method usually adopted in economic evaluations of public health policies and programs [39, 40] is based on the comparison of costs and health outcomes between groups of individuals allocated in intervention and control groups; therefore, in the present study, those who will have and have not experienced changes in the built environment will serve as intervention and control groups, respectively. Comparisons between individuals living in regions with different levels of the built environment for physical activity will also be conducted.

Analyses will be performed using the software MAXQDA Standard Educational, IBM SPSS Statistics version 24.0, Stata version 16.1, and NetLogo.

## Discussion

An international research project in partnership with Australian researchers was initiated in 2016 and 2017 to develop built environment indicators to inform physical activity promotion initiatives in Sao Paulo city [17], addressing the lack of studies in large metropolitan areas in Latin American countries. This initial study used cross-sectional data from the Health Survey of Sao Paulo 2015, which will comprise the baseline for this new prospective cohort study. Results indicated that adults who had access to two or more public open spaces (e.g., bike pathways, squares, and parks) in a radius of 500 m around their households presented a higher probability of leisure-time walking in comparison to individuals without access to those spaces around their households [17]. Moreover, people living up to 500 m from bike pathways had a higher probability of using the bicycle for transport in comparison to individuals without access to those structures nearby [16], and adults living in 500 m buffers with more mixed destinations adopted walking for transport more frequently than others [18].

However, longitudinal studies are required to avoid potential self-selection bias [19] and to obtain robust evidence on the effects of the built environment on health outcomes, especially in low- and middle-income countries undergoing rapid societal transition processes [12]. Well-established examples of longitudinal studies conducted in high-income countries are the “RESIDential Environment Study (RESIDE)” [20] and the “How Areas in Brisbane Influence Health and Activity (HABITAT)” [41], both in Australia, and the I-Connect [21], in the United Kingdom. More recently, the “Examining Neighbourhood Activity in Built Living Environments (ENABLE London study)”, which followed 1278 adults during 2 years, showed that an increase in walkability index, a decrease of distances between participants’ houses to parks, and an increase of public transportation access were associated with higher levels of walking [42].

Our study builds on the Health Survey of Sao Paulo 2015 with the main objective of obtaining and analyzing robust data to expand the scientific evidence that can inform public policy decision making towards healthy environments for physical activity promotion in low- and middle-income countries. We collect information on diverse built environment features from the areas where people live and work and their association with physical activity-related outcomes, especially leisure and transport ones, and estimate the potential population-level impacts of changes in the built environment, contributing to the planning and implementation of strategic changes in the urban design of Sao Paulo. Additionally, it will be possible to estimate the potential effects of COVID-19 restrictions on physical activity levels.

Another important planned outcome of our study will be the objective assessment of environmental characteristics in multiple time points, allowing the observation of changes in the built environment over the year and how they can affect health outcomes. Complementarily, built environment audits combining the MAPS method [35] and Google Street View will allow the observation of environmental features at scale, helping us to create and improve indicators that may be used to plan strategic actions to improve the population’s wellbeing. Processes for data extraction and estimation of built environment indicators using well-designed geoprocessing methods, like the ones applied by the “International Physical Activity and the Environment Network (IPEN)” [43], are essential to test hypotheses on the relationships between the built environment and leisure-time and transport-related physical activity and to provide reliable evidence to inform the development and implementation of physical activity promotion initiatives.

The IPAQ long version is widely adopted for the assessment of physical activity in studies that investigate the relationship between built environment and physical activity behavior (e.g., IPEN study [44]), and has been extensively used in studies in Latin American countries [27]. The IPAQ long version enables the investigation of each physical activity domain independently, a critical advantage for our study given that physical activity behavior in different domains may be affected by different environmental properties. The questionnaire was standardized to register physical activity in a typical week and it will allow the identification of changes throughout the follow-up period, particularly after COVID-19 pandemics. Additional questions regarding objectives, places, types, and duration of practices adopted by the participants were included for the face-to-face interviews, as well as questions on sedentary behavior in leisure and at work. The additional questions included did not affect the validity and reproducibility of the questionnaire, according to the reproducibility assessment performed with a sample of 43 individuals.

A potential disadvantage of IPAQ refers to its subjectivity and reliance on individuals’ memory, in addition to the overestimation of physical activity [45]. To counter these drawbacks our study will adopt an objective assessment of physical activity by accelerometry to collect movement patterns for 24 h per day over 1 week, including details on sleep, sedentary behavior, and light, moderate and vigorous physical activity [28–32].

Regarding nutritional status, self-reported weight and height were previously validated in a subsample of 856 participants of the 2015 survey [46]. Calibration coefficients for correction of weight, height, and BMI were also proposed in the study, considering sex, age, leisure-time physical activity, and exposure to cardiometabolic risk factors. The results obtained indicated that self-reported measurements of weight, height, and BMI were valid for telephone and face-to-face interviews, presenting intraclass correlation coefficients higher than 0.60 and, after calibration, an increase in the accuracy of self-reported measurements. Therefore, it will be possible to estimate with improved precision the prevalence of obesity in the follow-up study.

The results of the agent-based models and cost-effectiveness analysis of potential changes in the built environment can inform future policies aimed at promoting physical activity at scale, allowing those responsible for decision-making processes in infrastructure, health, and leisure programs to identify opportunities to provide, improve, and sustain environments that are conducive to wellbeing through physical activity, health and quality of life.

Another challenge that our study wants to address is the utilization of evidence by stakeholders involved in physical activity promotion. Thus, the engagement with actors and representatives of organizations and groups of the population has been extremely important in the study design and planning for implementation actions [37].

Finally, considering the lack of longitudinal studies in low- and middle-income countries, we believe that the ISA-Physical Activity and Environment will contribute with important results for the advancement of the knowledge on relationships between variables referring to the built environment nearby households and work, and leisure-time physical activity and active transportation.

## Data Availability

Not applicable because this paper describes the protocol of the study.

## Abbreviations

IPAQ: International Physical Activity Questionnaire
ISA: Health Survey of Sao Paulo
